# Policies and Programs for Prevention and Control of Diabetes in Iran: A Document Analysis

**DOI:** 10.5539/gjhs.v7n6p187

**Published:** 2015-04-16

**Authors:** Obeidollah Faraji, Koorosh Etemad, Ali Akbari Sari, Hamid Ravaghi

**Affiliations:** 1 Department of Health Management and Economics, School of Public Health, Tehran University of Medical Sciences, Tehran, Iran; 2 Department of Epidemiology, School of Health, Shahid Beheshti University of Medical Sciences, Tehran, Iran; 3 Department of Health Services Management, Health Management and Economics Research Centre, School of Health Management and Information Sciences, Iran University of Medical Sciences, Tehran, Iran

**Keywords:** diabetes, document analysis, Iran, policy analysis

## Abstract

Trend analysis in 2005 to 2011 showed high growth in diabetes prevalence in Iran. Considering the high prevalence of diabetes in the country and likely to increase its prevalence in the future, the analysis of diabetes-related policies and programs is very important and effective in the prevention and control of diabetes. Therefore, the aim of the study was an analysis of policies and programs related to prevention and control of diabetes in Iran in 2014.

This study was a policy analysis using deductive thematic content analysis of key documents. The health policy triangle framework was used in the data analysis. PubMed and ScienceDirect databases were searched to find relevant studies and documents. Also, hand searching was conducted among references of the identified studies. MAXQDA 10 software was used to organize and analyze data.

The main reasons to take into consideration diabetes in Iran can be World Health Organization (WHO) report in 1989, and high prevalence of diabetes in the country. The major challenges in implementing the diabetes program include difficulty in referral levels of the program, lack of coordination between the private sector and the public sector and the limitations of reporting system in the specialized levels of the program.

Besides strengthening referral system, the government should allocate more funds to the program and more importance to the educational programs for the public. Also, Non-Governmental Organizations (NGOs) and the private sector should involve in the formulation and implementation of the prevention and control programs of diabetes in the future.

## 1. Introduction

Islamic Republic of Iran, a country in the Middle East, has 75 million people, according to the last national census in 2011 (Statistical Center of Iran (SCI), October 24 2011). The urban and rural population of the country, in accordance with the same census, is 72% and 28%, respectively. Iran, similar to most developing countries, is in epidemiological transition. The Non-Communicable Diseases (NCDs) such as diabetes are replacing infectious diseases as the main causes of mortality and morbidity in developing countries. So that, 80% of NCDs deaths in the world occur in these countries (Golozar et al., 2011).

Diabetes is one of the most common NCDs worldwide. Seventy percent of people with diabetes lived in Low and Middle Income Countries (LMICs) in 2010 and the highest relative increase in the diabetes burden happens in Africa and the Middle East (Golozar et al., 2011). It is predicted to increase the diabetes prevalence in the developing countries up to 15% in the next 25 year. Most of this increase will occur in people aged 35 to 64 years old, whereas most people with diabetes in the developed countries are above the retirement age. So, the problem will more complicate in the developing countries (Wild et al., 2004).

First study about the diabetes prevalence in the country dates back to 1977. This study was conducted by the Iran’s Institute of Nutrition and Food Sciences on 6300 people. The results of the study showed increases of diabetes in employees compared to workers, as well as a high prevalence of diabetes in the desert areas of Iran. Also, the diabetes prevalence in the study was reported in children and adults 0.6–0.005 and 2–10 %, respectively (Azizi et al., 2003b).

Next study about the prevalence of diabetes was carried out by the research deputy of the Ministry of Health and Medical Education (MoHME) in 1990. The research results indicated the highest prevalence of diabetes in Tehran (capital of Iran). Also, the diabetes prevalence in the cities was more than the villages and in women more than in men. The study results were not reliable, because of collecting data from a questionnaire and lack of an indicator to measure blood glucose (Azizi et al., 2003a).

Systematic epidemiological studies were launched by the Endocrine Research Center and Institute of Food Technology, Shahid Beheshti University of Medical Sciences, on the people older than 30 years in Islamshahr (a city near Tehran) in 1993. The results of these studies showed that the diabetes prevalence was in women and men about 7.6% and 8.9, respectively, and also half of diabetic patients were unaware of their disease (Azizi et al., 2003c). Subsequent study based on the results of the Iran’s first survey of NCDs risk factors in 2006 showed that the diabetes prevalence was 7.7% (Delavari et al., 2005).

According to the latest studies in Iran, prevalence of diabetes and impaired glucose tolerance in people aged 25 to 70 years was 11.4 percent (4.5 million people) and 14.6 percent in 2011 respectively. Trend analysis in 2005 to 2011 showed 35 percent growth in the diabetes prevalence in Iran. The annual direct cost of diabetes in the country is estimated to be about 590$ million in 2009 (Esteghamati et al., 2014). Factors of increasing the diabetes prevalence in the country include low physical activity, rapid economic transition, urbanization, consumption of high-calorie foods and obesity (Farzadfar et al., 2012; Naghavi & Jafari 2007).

As stated by the international reports, it can be prevented at least 80 percent of heart disease, stroke and type 2 diabetes by eliminating the main factors causing these diseases (WHO, 2008). Considering the high prevalence of diabetes in Iran and likely to increase of its prevalence in the future, the analysis of the policies and programs for prevention and control of diabetes is very important and effective in reducing rates of prevalence of diabetes and its complications. Therefore, aim of the study was an analysis of policies and programs related to prevention and control of diabetes in Iran in 2014.

## 2. Methods

### 2.1 Study Design

**Figure 1:**
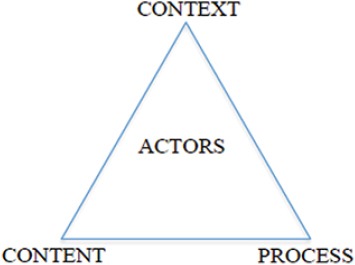
Walt and Gilson’s policy triangle framework

This study was an analysis of the diabetes policies and programs in Iran using deductive thematic content analysis of key documents. In this study, the health policy triangle framework was used in the data analysis. The Walt and Gilson’s policy triangle framework, that has been prepared specifically for study on health field, focuses on four fields of policy consist of content, context, process and actors who have a critical role in forming policies (Figure 1) (Walt et al., 2008).

### 2.2 Search Strategy and Document Finding

PubMed and ScienceDirect databases were searched to find studies about policies and programs for prevention and control of diabetes in Iran since 1989 (the first program of the WHO as for the prevention and control of diabetes) (Reiber & King, 1991) until July 2014. Key words to search were diabetes, policies, programs, processes, strategies, intervention and Iran. Also, hand searching was conducted among references of identified articles. In addition, reports of the international organizations like WHO, International Diabetes Federation (IDF) and World Bank (WB) were examined. Furthermore, internal websites such as the ministry of health website were searched for finding internal documents.

Aside from the original documents about the policies and programs of diabetes, the guidelines, survey reports and national meeting reports on the formulation and evaluation of the program were examined in the study. The included documents for the study were 21 documents: original documents (5), survey reports (2), guidelines (7), and national meeting reports on the formulation and evaluation of the program (7) (Table 1).

**Table 1:** List of analyzed documents in this study

**Original documents**	National diabetes prevention and control program (Delavari et al., 2004a) The comprehensive program for prevention and control of diabetes type II, Phase II, Run the program in the cities with over a million people (MoHME, 2010) National comprehensive program for prevention and control diabetes, the action plan of specialized care (Ravaghi et al., 2010) National program for prevention and control diabetes type II in urban areas (Alavi Nia et al., 2012) Management of specialized care program for diabetes (Ghotbi and Kermanchi, 2013)

**Survey reports**	A National Profile of Non-Communicable Disease Risk Factors in the I.R. Of Iran- Selected results of the first survey of Non-Communicable Diseases risk factor surveillance system of Iran, 2005 (Delavari et al., 2005) I.R. Iran Non-Communicable Diseases, Risk Factors Surveillance, 2008 (Asgari et al., 2008)

**Guidelines**	Principles of Disease Prevention and Care – Non-Communicable Diseases Surveillance system - National guidelines (Ghotbi et al., 2008) National diabetes prevention and control program for physicians (Delavari et al., 2004f) National diabetes prevention and control program for nurses (Delavari et al., 2004e) National diabetes prevention and control program for technicians (Delavari et al., 2004g) National diabetes prevention and control program for dietitians (Delavari et al., 2004b) National diabetes prevention and control program for health workers (Delavari et al., 2004c) National diabetes prevention and control program for laboratory technicians (Delavari et al., 2004d)

**National meeting reports**	National meeting report on the comprehensive diabetes program (Mashhad, October 2011)(Ghotbi, 17 October 2011) National meeting report on the comprehensive diabetes program (Kashan, January 2012) (MoHME, 1 January 2012) National meeting report on the comprehensive diabetes program (Kerman, April 2012) (MoHME, 14 April 2012) National meeting report on the comprehensive diabetes program (Yazd, March 2013) (MoHME, 3 March 2013) Workshop report about evaluation and quality improvement of specialized care program for diabetes (Bandar Abbas February 2014)(Ghotbi, 17 Febuary 2014) Analytical performance report of the urban phase of the comprehensive diabetes program, 2012 (Ghotbi, 2012) Performance report of the urban phase of the specialized care program for diabetes, 2013 (Ghotbi, 2013)

### 2.3 Data Analysis

Documents using thematic content analysis were classified, described and analyzed. The documents were sorted from old to new chronology and were examined several times by the research team. Afterwards, primarily coding of all documents was conducted. Next, Initial codes were put on the main themes based on the study’s conceptual framework by the first researcher. After that, the researchers agreed on the data analysis process and finally the coding instruction was prepared. Then, all documents were coded and themes were re-classified by the researchers using the coding instruction. After the researchers performed the coding, the codes were reviewed by the research team. And finally, the consensus was generated on the final codes and themes. MAXQDA 10 software was used to organize and analyze data.

## 3. Results and Discussion

### 3.1 Content

A pilot project for prevention and control of diabetes was implemented in some areas of the country in 1992. The project results were evaluated in 1993. The results of the evaluation include the non-standard method for screening, the high costs of screening and surveillance for each patient, lack of adequate equipment in the health network system to implement the program and the low prevalence of diabetes in the rural areas. The results were lead to stopping the program (Delavari et al., 2004a).

**Figure 2:**
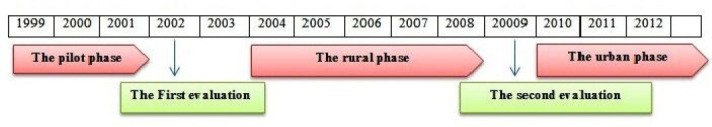
Timeline of the national diabetes prevention and control program in Iran

The national diabetes prevention and control program was implemented in the pilot phase in 17 medical universities for people over 30 years and pregnant females in 1999 to 2001. After the pilot implementation, the program was launched in two phases (rural phase and urban phase) in 2004 and 2010 respectively (Figure 2). The target population of the both phases is the high-risk adults older than 30 years and pregnant women. The screening in the rural phase was active, but the screening in the urban phase is passive and opportunistic (Azizi et al., 2003a; Alavi Nia et al., 2012).

The overall goal of both of the phases was prevention and control of diabetes and its complications. The specific objectives, strategies and outcomes of the program are shown in table 2 (Alavi Nia et al., 2012; Delavari et al., 2004a). Although the goals and strategies of the program were largely consistent with the objectives of the world diabetes program in 1989 (Reiber & King 1991) and the global action plan for prevention and control of NCDs in 2013 (WHO, 2013), collaboration with other stakeholders such as the private sector and NGOs and well as the important role of social determinants in preventing diabetes through creation of health–promoting is not mentioned in the program.

**Table 2:** Objectives, strategies and outcomes of the program

**primary**	- to reduce the incidence and prevalence of diabetes type II - to reduce the prevalence of modifiable risk factors to the condition (obesity, physical inactivity, unhealthy diet, etc.)

**Objectives secondary**	- to prevent, reduce and delay the short and long-term complications of diabetes

**tertiary**	- to reduce and delay the incidence of disability and premature death due to the diabetes complications - to reduce loss of life of people with diabetes

**Common strategies in both phases**	- to gain the support of policy makers - to determine needed resources and the minimum standards for care of diabetes - helping the health centers to develop and improve needed laboratory equipment - help to develop the reference laboratories for conducting the quality control tests related to diabetes - help to provide needed medicines, equipment and material for control, monitoring, and self-monitoring of diabetes - to support of the researches related to diabetes - to screen employees of the trade unions and governmental and NGOs in the urban areas - to screen and increase awareness of people about diabetes in the special days and places

**Added strategies to the urban phase**	- to educate and inform the general population - obtaining the government participation in informing society and empowering patients and their families - to strengthen the referral system in diabetes care - appropriate and timely treatment of diabetic patients - to strengthen intra and inter-sectoral coordination in providing services to the referred patients - to enable the diabetes team at different levels of care - to promote the surveillance systems, monitoring and evaluating of diabetes care - to increase the international participation in implementing the program

**Outcomes**	- to reduce economic costs due to diabetes and its complications - to reduce disabilities due to diabetes and its complications - to reduce premature mortality due to diabetes and its complications - to improve quality of life and to increase life of patients with diabetes

### 3.2 Process

#### 3.2.1 Agenda-Setting

The National Committee for Diabetes was formed in summer 1996 after releasing the results of many studies which showed the high prevalence of diabetes in the country. The objective of the committee formation was the provision of the national diabetes program and the training patterns for the public and providers. The committee members presented a program with the overall goal of prevention and control of diabetes. The MoHME, in coordination with the National Committee for Diabetes, held six workshops related to diabetes for doctors, nurses and nutritionists in 1997 and 1998. Afterward, the program was implemented as a pilot plan in 1999 (MoHME, 2010; Azizi et al., 2003a).

The reasons into consideration diabetes in Iran can be the WHO report in 1989 and the obligation of member states to plan for prevention and control of diabetes in the countries (Reiber & King, 1991), high prevalence of diabetes in the country in many researches (Azizi et al., 2003a), the commitment and insistence of the health minister for implementing the diabetes program in 1996 (Delavari et al., 2004a) and allocating a budget from taxing on sugary drinks to diabetes program approved by the Parliament of Iran (PoI, 2007) which provided valuable funds to start the program.

#### 3.2.2 Formulation

The program formulation was based on the WHO instructions and guidelines. Also, the regulatory requirements such as the fifth development plan of the country as well as the capacity of the medical universities and willingness to implement the program among the board of trustees of the medical universities were considered (Ghotbi & Kermanchi, 2013). Despite considering the important issues such as the capacity and coordination within the medical universities in formulating the urban phase, but the experiences of successful countries have been used very little.

In formulating the rural phase, the diabetes workshops were held for the chief executives of medical universities in the country for 4 days and more than 270 people. At these meetings, the duties of diabetes team, the educational materials for diabetes, and the educational texts for the health workers and the physicians were prepared. Also, the workshops were held for the physicians, nurses, nutritionists across the country (Delavari et al., 2004a).

With regard to the increased prevalence of diabetes in the metropolitans, planning to control of diabetes was begun in the cities with populations over one million people since 2010. The necessary changes were applied in the previous program to perform it in the cities. This program was designed given the breadth and diversity of the target population in the metropolitans. After several meetings with the officials and experts of the selected medical universities, feedbacks about the program were reviewed. Meanwhile, the educational literatures for service providers were revised and also a set of executable instructions was prepared. After making the necessary changes in the program, the final program was sent to the medical universities and the program funds were distributed among the medical universities (MoHME, 2010).

The training workshops were held by the Center for Non-Communicable Disease Control for the program providers in 2010. Also, the prerequisites for the program implementation were prepared by the medical universities and the personnel responsible for implementing the program were trained (Alavi Nia et al., 2012).

#### 3.2.3 Implementation

The national diabetes prevention and control program was integrated into the primary health care of the health system in October 2004 (Alavi Nia et al., 2012; Ravaghi et al., 2014).The program was not implemented in the cities. The reasons for it include lack of the coherent and comprehensive health network and active network system needed to provide the services in the cities as well as lack of enough fiscal resources to implement the program in the whole country (MoHME, 2010).

The rural phase was run by the health workers in the rural health centers. The screening for all adults aged 30 years and older of the villages was implemented in two rounds in 2004 and 2008. But, the screening was done once a year for people with pre-diabetes. The medical universities were responsible for implementing the program in the rural areas. They could collaborate with the private sector if needed (Delavari et al., 2004a).

The urban phase was designed in 2009. The program at the urban level is defined in three levels include the first level (diabetes units (urban health centers or private clinic) for the screening and patient care), the second level (specialized hospitals) and the third level (sub-specialized centers) (Ravaghi et al., 2014).

After the evaluation and redesign of the program, it was implemented in Tehran and five metropolitans of the country in 2010. The other three medical universities were added to the program and meanwhile, the second level of specialized services was launched through collaborating with the health and treatment deputies of the medical universities. Six and eight other universities joined to the program in 2011 and 2012 respectively (Ghotbi & Kermanchi, 2013).

The main criterion for joining the medical universities in the urban phase is the collaboration between health and treatment deputies for the comprehensive and uninterrupted use of the integrated health services (MoHME, 1 January 2012). To write the operational program is the responsibility of the medical universities. But the program must be approved by the MoHME. The operational program should be based on the size of the diabetes problem as well as the capacity of executive system in the provinces. The funds will be distributed to the medical universities after the operational program approval for a three-month period. Afterward, the funds for the program will be placed at the disposal of the universities based on the performance of three-month period (Ghotbi & Kermanchi, 2013; Ravaghi et al., 2010).

In addition to the national diabetes committee, the medical universities have an advisory committee for diabetes. This committee is responsible for decision-making, the approval of the operational program and allocating funds for implementing the diabetes program at the university level. All diabetic patients, which are under care at the health centers, are referred to the hospital to assess the chronic complications once yearly. In some cases, the diabetes units are run by the private sector (Alavi Nia et al., 2012).

#### 3.2.4 Evaluation

The program was evaluated for the first time using a questionnaire at the six medical universities in 2002. The evaluation results showed higher prevalence of diabetes in the cities than in the villages, the highest prevalence in the industrial urban areas, higher prevalence in women than in men as well as the program success in the rural areas and high consent of implementing the program in the rural population (MoHME, 2010; Farzadfar et al., 2012). The second evaluation of the program was conducted in 2009. The evaluation results showed the failure in the referral system and complications control. Also, there wasn’t a good performance in the control of complications except in the retinopathy control. The evaluation results were used to review and to modify the urban program (Ghotbi & Kermanchi, 2013; Delavari et al., 2009). Besides these evaluations, two rounds of the Non-Communicable Disease risk factors survey were conducted in 2005 and 2008 which the results are shown in Table 3 (Asgari et al., 2008; Delavari et al., 2005).

**Table 3:** Results of two rounds of Non-Communicable Disease risk factors survey in 2005 and 2008

Indicator	Indicator of Report 2005 (percent)			Indicator of Report 2008 (percent)		
	
	Male	Female	Total	Male	Female	Total
**The mean fasting blood glucose (mg/dl)**	94.6	95.1	94.9	89.51	88.96	89.24
**Percentage of people with high blood glucose (> 126 mg/dl)**	5.6	6.1	5.9	9.41	9.97	9.69
**The mean serum cholesterol (mg/dl)**	194.6	200.2	197.6	181.4	189.7	185.5
**Percentage of people with high blood cholesterol (> 200 mg/dl)**	41	46	43.7	35.1	45.12	40.04

The program evaluation in the urban phase is conducted by the process evaluation indicators at the university level every six months. The analytical performance reports are prepared by the medical universities, according to pre-prepared forms. These reports are regulated jointly by the program’s health experts at the health deputy and the program’s executive focal points at the treatment deputy of the medical universities. Evaluation of the program effectiveness is conducted through periodic evaluations during the specific periods as well as the external evaluation methods by the headquarters of the ministry of health.

The program effectiveness indicators include the percentage of people aged 30 years and older covered by the program, the incidence rate of diabetes in people aged 30 years and older, the prevalence rate of diabetes in people aged 30 years and older, the incidence rate of disability due to the diabetes, the prevalence rate of disability due to the diabetes, the incidence rate of gestational diabetes, as well as the rate of optimal glycemic control in the diabetic patients covered by the program, the hypertension prevalence in the diabetic patients and the annual incidence of the chronic complications in diabetic patients covered by the program which These indicators are calculated by age, sex and geographic area (Ravaghi et al., 2010).

### 3.3 Context

The contextual factors affecting the program implementation in the country include the political and administrative factors, the economic and financial factors and the social and cultural factors.

The political and administrative factors include the capabilities of the health network system in the implementation of the program, the possibility of providing the program services at the lowest level of the health network system, the existence a system to collect and to report health information from the lower levels to the higher levels of the health system (Alavi Nia et al., 2012; Delavari et al., 2004a), difficulty in the referral levels of the diabetes program, the shifting of staff responsible for the program implementation (MoHME, 14 April 2012; Ghotbi & Kermanchi, 2013), lack of adequate cooperation between health and treatment deputies in the medical universities, lack of coordination between the private sector and public sector, limitations of the data entry and reporting system in the specialized levels of the program and shortage of manpower and skills needed to implement the program (Alavi Nia et al., 2012; Ghotbi, 2013). The results about the limitations of the data entry and reporting system and manpower and skills shortages is along with the results of the study of Ravaghi and et al. (2014).

The economic and financial factors include lack of funding for optimal implementation of diabetes program, a lack of health insurance coverage for most of diagnostic services, particularly advanced services, lack of equal and constant access to the treatment requirements at the different times of the year, the unavailability of some types of insulin at the sometimes, lack of access to syringes with high quality, lack of diagnostic facilities in the second level of the program, low number of equipped laboratories in the rural health centers and lack of access and high costs of the self-monitoring facilities like glucometer (Ghotbi, 17 October 2011; Ghotbi, 2012).

The social and cultural factors include lack of harmony and cooperation of the cultural, social and economic streams in the country, low level of health literacy in the country, lack of access to healthy living choices, culturally and economically (Ghotbi, 17 Febuary 2014).

### 3.4 Actors

Participants in formulating the rural phase included the national diabetes committee members, the representatives of the medical universities; Department of Endocrinology and Metabolic of Center for Non-communicable Disease Control, Center for Network Development and Health Promotion, Bureau of Population & Family Health, and Office of Community Nutrition Improvement of Ministry of Health and Medical Education (Delavari et al., 2004a). Participants in formulating the urban phase included Department of Endocrinology and Metabolic of Center for Non-communicable Disease Control, Office of hospital administration and Clinical Service Excellence, Endocrinology & Metabolism Research Institute of Tehran University of Medical Sciences, the chancellors and vice- chancellors of the medical universities, Iranian Society of Nephrology, the financial director of treatment deputy, the general manager of Center for Non-communicable Disease Control and the program experts at the medical universities (Alavi Nia et al., 2012).

Although the more stakeholders involved in the urban program formulation compared to the rural program, but like the rural program, most of them were internal stakeholders and the key stakeholders outside of the health sector did not participate in the program formulation. As the results of studies show a low collaboration among various stakeholders in the policy formulation in developing countries (Kilic et al., 2015; Mendis & Chestnov, 2013).

## 4. Conclusions

Despite the attention to the diabetes problem and the beginning of the program and its integration into the health network system about 10 years ago, the prevalence of diabetes and its complications is rising in the country. Therefore, it is required that the health officials and policy makers focus more on the prevention issues and reducing risk factors in the society. The study limitations include the only use of the policy documents in the policy analysis as well as lack of using NGO’s documents in the policy analysis.

Given the large population in the cities were covered by the diabetes urban program in the last four years, It is hoped the program will reduce the diabetes incidence and prevalence in the country. To achieve this, the physicians should use the guidelines regarding glycemic control for the treatment of the diabetic patients. The revision of the insurance contract as well as increase of insurance premium is recommended for the patients who did not attempt to control their blood sugar.

Also for increasing the program effectiveness, in addition to strengthen the referral system, the government should allocate more funds and importance to the educational programs. Furthermore, NGOs and the private sector should contribute to the formulation and implementation of the diabetes prevention and control programs in the future. In addition, the government should provide adequate medical facilities for prevention and control of diabetes in the country. And also, with regard to the important role of the community members and patients in the program success and the patient- oriented nature of diabetes, they should pay more attention to the their health through proper nutrition, adequate physical activity and awareness of the physical health status.

## Ethical Considerations

Ethical issues (Including plagiarism, double publication and/or submission, redundancy, etc.) have been completely observed by the authors.
